# What Is New in Thyroid Cancer: The Special Issue of the Journal Cancers

**DOI:** 10.3390/cancers12103036

**Published:** 2020-10-19

**Authors:** Efisio Puxeddu, Giovanni Tallini, Roberta Vanni

**Affiliations:** 1 Department of Medicine, University of Perugia, 06123 Perugia, Italy; efisio.puxeddu@unipg.it; 2 Department of Experimental, Diagnostic and Specialty Medicine, University of Bologna School of Medicine, 40126 Bologna, Italy; giovanni.tallini@unibo.it; 3 Department of Biomedical Sciences, University of Cagliari, 09124 Cagliari, Italy

**Keywords:** thyroid cancer, microcarcinoma, intratumoral heterogeneity, thyroid genomics, thyroid transcriptomics, predictive biomarkers, cancer epidemiology, thyroidectomy, MALDI-MSI, lncRNAs

## Abstract

The incidence of thyroid cancer has increased over the past 3 to 4 decades. Nonetheless, the mortality from thyroid cancer has remained stable. The thyroid gland may develop nodules encompassing several types of cell proliferation, from frankly benign to very aggressive forms with many intermediate challenging variants. For this reason, there is growing interest in evaluating thyroid nodules from many points of view, from the clinical to the molecular aspects, in the search for innovative diagnostic and prognostic parameters. The aim of this Special Issue was to provide an overview of recent developments in understanding the biology and molecular oncology of thyroid tumors of follicular cell derivation and their repercussions on the diagnosis, prognosis, and therapy. The contributions of many experts in the field made up a Special Issue of Cancers journal, that focusing on different aspects, including mechanistic and functional facets, gives the status of art of clinical and biological perspectives of thyroid cancer.

The incidence of thyroid cancer has dramatically increased in the last 40 years, due to both an improved detection of small (<2 cm) thyroid nodules by neck ultrasonography and a true escalation in thyroid cancer incidence. In spite of this dramatic increase, the vast majority (more than 85%) of thyroid cancer patients are cured by initial treatment and mortality is stably low. This claims for the need of precise parameters able to distinguish between indolent and clinically aggressive nodules, to avoid overtreatment and to accurately identify those tumors that require more aggressive therapy. Molecular biology has been of great benefit in this respect, and new classifications, taking into account molecular parameters, have recently been introduced, defining new subclasses of thyroid tumors. The aim of this Special Issue was to provide an overview of recent developments in understanding the biology and molecular oncology of thyroid tumors of follicular cell derivation and their repercussions on the diagnosis, prognosis and therapy.

Contributions to all aspects related to the topic were expected with particular interest for articles characterized by mechanistic and functional insights from a cellular and molecular biological perspective.

We think that the goal was hit with the inclusion of twenty original research articles, and eight reviews, which passed a very strict reviewing process, were timely and covered many important aspects in the field.

In detail, five papers on genetics and genomics of thyroid cancer were published. Boufraqech and Nilubol reviewed the major molecular alterations of the genome, epigenome, transcriptome, proteome and metabolome found in all subtypes of thyroid cancer, and they reasoned on the great translational potential of multi-omics studies to improve patient outcome [1]. Acquaviva et al. evaluated by NGS the *BRAF* exon 15 status in thyroid tissue samples adjacent to papillary thyroid carcinoma (PTC), with the aim of better understanding whether potential precursor lesions are associated with its development. Interestingly, their data indicated that a mutagenic process affecting *BRAF* exon 15 takes place in a subset of thyroid glands that develop *BRAF* p.V600E mutated PTCs. While the carcinoma develops only if the p.V600E mutation occurs, its development is accompanied by other exon 15 mutations [2]. Fugazzola et al. reviewed the data in favor of the occurrence of intratumoral heterogeneity in PTC and reasoned on its impact on prognosis and on the response to targeted therapy [3]. Moretti et al. described a wide expression of the Aryl Hydrocarbon Receptor in all follicular-derived thyroid carcinoma histotypes and demonstrated its role in conferring a more aggressive phenotype to thyroid cancer cells, by contributing to the onset of an immune-tolerant microenvironment and promoting epithelial-mesenchymal transition [4]. Minna et al. analyzed whether the cross-talk between thyroid tumor cells and Cancer Associated Fibroblasts (CAFs) also occurred in human thyroid cancer, and whether senescent thyroid cells might be potentially involved in this process. Interestingly, they showed that CAFs and senescent thyroid cancer cells are both present at the tumor invasive front of *BRAF*-driven thyroid carcinomas. Thus, their data highlighted the possibility that those cell types represented additional players of the metastatic process in thyroid cancer [5].

The Special Issue is also enriched by two methodological articles. In the first paper, Ylli et al. analyzed the utility of digital PCR technique in detecting *BRAF^V600E^* and *TERT* promoter mutations (*C228T* and *C250T*) in thyroid cancer tissue samples. Interestingly, microfluidic digital PCR allowed for specific, sensitive and rapid detection of *BRAF^V600E^* and *TERT* C228T mutations in thyroid tissue samples and could be used for quantification of mutant alleles in an easy and cost-effective manner [6]. In the second paper, Capitoli et al. applied, for the first time, Matrix-Assisted Laser Desorption/Ionization (MALDI) Mass Spectrometry Imaging (MSI) on real thyroid Fine Needle Aspirations (FNAs) to test its possible complementary role in routine cytology in the diagnosis of thyroid nodules. Firstly, they designed a statistical model based on the analysis of Regions of Interest (ROIs), according to the morphological triage performed by the pathologist. Successively, the capability of the model to predict the classification of the FNAs was validated in a different group of patients on ROI and pixel-by-pixel approach. Results were very promising and highlighted the possibility to introduce MALDI-MSI as a complementary tool for the diagnostic characterization of thyroid nodules [7].

Appetecchia et al. presented the result of a multicenter study addressing the epidemiological characteristics, disease conditions and clinical outcome of patients with simultaneous medullary thyroid carcinoma/papillary thyroid carcinoma. The obtained results supported the notion that in those cases the priority should be given to the management of medullary thyroid carcinoma (MTC), that calcitonin should always be included in the workup of multinodular goiter, even in the presence of cytology positive for PTC, and that early diagnosis of MTC is still an unmet need. Furthermore, the importance of genomics for early diagnosis, prognosis and therapy of these cancers was discussed [8].

Six papers addressing pathologic or molecular pathologic issues were also included. Choi et al. evaluated the cytomorphologic features of PTC related to lymph node metastasis in preoperative thyroid FNAC specimens and revealed that atypical histiocytoid cells and multinucleated giant cells were independent predictors of lymph node metastasis in patients with PTC [9]. Tallini et al. measured the origin of papillary thyroid microcarcinoma (mPTC) with respect to the thyroid capsular surface: mPTCs arise at a median distance of 3.5 mm below the surface of the thyroid gland, independent of the gland dimension. An easy four-group risk subdivision of mPTC based on tumor size and location that can be applied before surgery was proposed by the authors. Based on their findings, nodules ≥5 mm, attached to the thyroid capsule and with irregular margins (group A tumors of this study),are most likely associated with *BRAF^V600E^*, lymph node metastases, intermediate ATA recurrence risk, aggressive histotype (tall cell) and invasive features and need a more intense workup. Conversely, most other mPTCs, and particularly those that are small and non-subcapsular (group D tumor of this study) may safely avoid aggressive preoperative screening and immediate surgical treatment [10]. Xu and Ghossein compared and summarized the key prognostic pathologic characteristics of thyroid carcinoma utilized by the most influential clinical and pathologic guidelines and provided a comprehensive review on the definitions and prognostic impacts of these pathologic parameters [11]. Jeon et al. investigated the *CCND1* rs9344 (G870A) polymorphism and the expression profiles of wild-type *CCND1a* and shortened oncogenic isoform *CCND1b* at the mRNA and protein levels in thyroid tumors. They suggested that cyclin D1b overexpression, also correlated to the genotype AA of rs9344, can be used as a diagnostic and predictive biomarker in thyroid tumors and may be functionally involved in the development and progression of the disease [12]. Chakladar et al. analyzed the co-dysregulation of immune-associated (IA) and cancer-associated (CA) genes in the different subtypes of PTC using a multi-scale approach, on data provided by The Cancer Genome Atlas (TCGA). They discovered that the dysregulation profiles of classical PTC (CPTC) and the tall cell variant PTC (TCPTC) are similar while that of the follicular variant PTC (FVPTC) are different. In detail, CA genes *MUC1*, *FN1* and S100-family members were the most clinically relevant in CPTC, while *APLN* and *IL16*, both IA, were clinically relevant in FVPTC. RAET-family members, also IA, were clinically relevant in TCPTC [13]. Boos et al. performed a comprehensive miRNA profiling to identify transcriptomic changes on the miRNA level in PTC. Interestingly, some miRNAs appeared to predict outcome in PTC and to be involved in TC-morphology in PTC [14].

Two papers addressed the issue of the radiation effect in thyroid cancer pathogenesis. In the first paper, Zupunski et al. expanded a previously published population-based case-control study on subjects exposed to iodine-131 (131I) from Chernobyl fallout at age ≤18 years. A statistically significant linear quadratic dose-effect association between thyroid cancer and 131I thyroid dose in the range up to 5 grays (Gy) was found. Self-reported personal history of benign nodules, any thyroid disease except thyroid cancer, family history of thyroid cancer, increased body mass index and deficient stable iodine status at the time of the accident were statistically significant risk factors for thyroid cancer after adjustment for thyroid 131I dose effect. Subjects who received stable iodine supplementation in the years after the accident had a significantly lower 131I-related risk of thyroid cancer. Thus, this study adds important pieces of knowledge for thyroid cancer prevention, and for further improvement of medical surveillance in the affected populations [15]. In the second paper, Suzuki et al. reviewed the literature about the identification of oncogenic alterations, particularly gene rearrangements, and the existence of radiation signatures in radiation-induced thyroid cancers [16].

Three papers addressed different aspects of differentiated thyroid carcinoma treatment. Geron et al. aimed to contribute more evidence regarding the long-term outcome of PTC patients treated with hemithyroidectomy, searching for possible prognostic factors and differences with similar stage patients treated with total thyroidectomy. Analyzing a retrospective casuistry composed by a 95% of low risk patients, either TNM stage I and low ATA risk, no significant differences were found in the rate of persistent and recurrent disease, overall mortality, and disease status at last visitwhen comparing the hemi- and total thyroidectomy groups. The conclusion was that for properly selected low-risk PTC patients, hemithyroidectomy is a safe treatment option with a favorable long-term outcome [17]. Aashiq et al. reviewed the proposed mechanisms for RAI refractivity and the management of RAI-refractive metastatic, recurrent thyroid cancer. Moreover, they described novel targeted systemic agents that are in use or under investigation for RAI-refractory disease, their mechanisms of action and their adverse events [18]. Crispo et al. provided an overview on the current knowledge concerning the mechanisms leading to resistance to *BRAF* inhibitors in human thyroid carcinomas and discussed the potential therapeutic strategies, including combinations of *BRAF* inhibitors with other targeted agents, which might be employed to overcome drug resistance and potentiate the activity of single agent *BRAF* inhibitors [19].

Five papers addressed the prognosis of differentiated thyroid carcinoma. Medas et al. evaluated the recurrence rate of DTC in a high-volume, regional referral center for thyroid carcinoma. In their hands, five-year disease-free survival was 95.8% in patients with negative lymph nodes and 73.7% in patients with metastatic lymph nodes, and 96.1% in patients with a microcarcinoma and 91.5% in patients with tumor >1 cm [20]. Collina et al. evaluated the impact of AXL on thyroid cancer progression. In detail, they evaluated AXL expression in human PTC and analyzed the signal transduction pathways mediating AXL actions in thyroid cell lines, including its detrimental effect on NIS. Their results suggested that AXL may be used as new risk assessment tool defining PTC patients at risk of RAI refractoriness, tumor recurrence or progression. Furthermore, AXL appeared to be a potential therapeutic target for RAI-R diseases [21]. Gąsior-Perczak D et al. assessed whether the coexistence of both somatic *BRAF^V600E^* and germline *CHEK2* mutations in PTC is associated with a poorer disease course. Analysis of clinicopathological features, primary treatment responses or disease outcome in a PTC cohort characterized for the two mutations, allowed them to conclude that the coexistence of both mutations is not associated with more aggressive clinicopathological features of PTC, poorer treatment response or disease outcomes [22]. Kim et al. applied non-negative matrix factorization-based deconvolution to publicly available gene expression profiles of thyroid cancers in the Cancer Genome Atlas (TCGA) consortium. Among three metagene signatures identified, two signatures were enriched in canonical *BRAF*-like and RAS-like thyroid cancers with up-regulation of genes involved in oxidative phosphorylation and cell adhesions, respectively. The third metagene signature representing up-regulation of immune-related genes further segregated *BRAF*-like and *RAS*-like PTCs into their respective subgroups of immunoreactive (IR) and immunodeficient (ID), respectively. Thus, the immune-related metagene signature could classify PTCs into four molecular subtypes, featuring the distinct histologic type, genetic and transcriptional alterations and potential clinical significance [23].

Finally, five papers addressed important issues in the frame of anaplastic thyroid carcinoma. Pelecchia et al. analyzed the expression of lncRNAs in anaplastic thyroid carcinoma (ATC) and controls. They focused on the lncRNA Prader Willi/Angelman region RNA5 (PAR5) that was found to be significantly and constantly decreased in ATC, but not in PTC tissues. Interestingly, the restoration of *PAR5* reduced proliferation and migration rates of two ATC-derived cell lines while it appeared to directly interact with EZH2, reducing its binding on the E-cadherin promoter [24]. Simões-Pereira et al. analyzed the clinical presentation, therapeutic modalities and outcomes, as well as the prognostic factors influencing disease specific survival (DSS), in a homogenous population of ATC followed at a single institution. They showed that most of the patients join reference centers at advanced stages of disease and multimodality treatment may offer the best chances for prolonging survival [25]. Ravi et al. performed whole exome sequencing and the RNA-sequencing of fourteen cases of ATC to delineate copy number changes, fusion gene events, and somatic mutations. Several alterations were detected, including among others *CCNE1*, *CDK6* and *TWIST1* amplifications, 21 gene fusions, mutations of *TP53*, *TERT* promoter and ATM. The obtained results shed new light on the tumorigenesis of ATC and showed that a relatively large proportion (36%) of ATCs harbor genetic events that make them candidates for novel therapeutic approaches [26]. Ljubas et al. summarized the results of phase II clinical trials of targeted therapy in ATC, providing an overview of efficacy and safety profiles. The result was a nice review of up-to-date treatment approaches for ATC or ongoing clinical trials [27]. Malfitano et al. reviewed the results of the studies evaluating the efficacy of Oncolytic Viruses alone or in combination with other treatment options in ATC. In particular, their potential therapeutic combinations with multiple kinase inhibitors or immune checkpoint inhibitors were discussed [28], and a comment by Dr Albert [29] from the Rigvir company highlighted the interest for Virotherapy in the treatment of cancer.
